# Dynamic Fracture Resistance under Plane Strain Conditions of High-Density Polyethylene Nanoclay Composites

**DOI:** 10.3390/polym15040813

**Published:** 2023-02-06

**Authors:** H. R. López-Cabrera, U. Figueroa-López, A. C. Taylor, A. Guevara-Morales

**Affiliations:** 1 Escuela de Ingeniería y Ciencias, Tecnologico de Monterrey, Atizapán de Zaragoza 52926, Mexico; 2 Department of Mechanical Engineering, Imperial College London, South Kensington Campus, London SW7 2AZ, UK

**Keywords:** polymer nanocomposites, rapid crack propagation, dynamic fracture resistance, high-speed double torsion test, crazing, montmorillonite nanoclays

## Abstract

Polymer nanoclay composites have received significant attention due to their substantially enhanced mechanical, thermal and barrier properties. However, the effect of these nanoclays on the dynamic fracture resistance of a polymer matrix during fast fracture events has not been documented. In this study, the effect of nanoclay addition on the rapid crack propagation (RCP) resistance of high-density polyethylene (HDPE) was investigated through the high-speed double torsion test. Results showed that the addition of 1, 3, and 5% of nanoclays improved the dynamic fracture resistance under the plane strain conditions ($G_{d1}$) of HDPE up to 65%. An increase in the storage and loss modulus, and a decrease in crystallinity and melt flow index with nanoclay content was also found. Although the presence of agglomerates can hinder the enhancement of $G_{d1}$ as it promotes agglomerate fracture and debonding, the increase in energy consumption through fibrillation and crazing promoted by the nanoclay prevails, suggesting that the nanoclay’s toughening effect that has been extensively reported under quasi-static and impact tests, is also present under RCP conditions, and that the HDPE nanocomposites could be used in applications in which RCP must be prevented.

## 1. Introduction

High-density polyethylene (HDPE) is one of the most widely used commodity thermoplastics, considered frequently for materials substitution because of its excellent availability and recyclability [1]. However, to match the profile of typical engineering thermoplastics, a substantial enhancement in its mechanical properties is required (e.g., modulus and impact strength). It has been reported that the use of nanofillers can significantly improve the stiffness, strength, and toughness of polymers, e.g., [2]. The use of organo-silicate clays has been extended to various polymers’ matrices such as polyolefins, polyamides, polyurethanes, epoxy resins, amongst others [3]. The ability of the organo-silicate clays to separate into individual platelets, together with their similar size to that of polymer molecules (which allows intimate mixing and chemical bonding) [4], and the possibility of modifying their surface chemistry through ion exchange reactions, make these clays ideal as nanofillers [3]. Other advantages are their availability, low cost, high thermal inertness, and environmentally friendly characteristics [5].

Regarding the toughening effect of nanoclays, Ou et al. [6] reported a 78% increase in the impact strength when a 5 wt% of silica nanoparticles were added to a PA6 matrix. Kinloch & Taylor [7] reported that the fracture energies of epoxy–clay nanocomposites increased at low volume fractions of clays, but decreased when the concentration of clays was increased further. They identified debonding and plastic deformation around the nanoclays as the main toughening effect as well as crack deflection. Similarly, Akbari & Bagheri [8] concluded that the nanoclays in an epoxy matrix act as shear bands initiation sites, improving the energy absorption through plastic deformation prior to fracture, and thus improving the fracture toughness of the material. For the case of thermoplastic olefins, Deshmane et al. reported [4] that their reinforcement with nanoclay increases their modulus and yield strength while retaining their impact strength. Hedayatnasab et al. [9] reported that the notched impact strength of polypropylene increased up to 62% at both room and high temperatures, with the addition of nanoclays. Mohagheghian et al. [10] studied the quasi-static and dynamic behavior of linear low-density polyethylene (LLDPE) nanocomposites filled with nanoscale carbon black and nanoclays. An improvement was observed in the energy absorbing capability of LLDPE under quasi-static tensile loading when nanofillers were added. However, under impact loading, filled and unfilled LLDPE performed similarly. Tanniru et al. [1] reported that the addition of nanoclays to a HDPE matrix decreases its impact strength in the −40 to 70 °C temperature range. This detrimental effect was associated with the crystal structure and weak interfacial interaction between the filler and the polymer matrix. Despite the numerous works regarding the toughening effect of nanoclays, little data have been published to date on the dynamic response of PE-based nanocomposites that are not based on Izod, Charpy, or similar impact experiments. Although these tests continue to be used in industry as an economical quality control method to assess the notch sensitivity and impact toughness of polymers [11], it has long been recognized that dynamic fracture is a more complex phenomenon that requires a deep analysis of crack evolution, and of the relationships between the material structure and morphology and the different energy dissipation mechanisms.

Because of the ductile/brittle transition behavior of most semi-crystalline polymers at low/high strain rates, understanding the effect of nanoclay addition on the impact behavior of these polymers under high-strain rate events is fundamental. Of particular interest for HDPE is a phenomenon known as rapid crack propagation (RCP), a dynamic fracture event in which a crack propagates through a structure at speeds higher than 100 m/s and that should be considered in the design of pipelines [12], geomembranes [13], and other applications in which the rapid propagation of a crack can result in a catastrophic event. Therefore, the aim of this study is to evaluate the effect of reinforcing HDPE with organo-silicate nanoclays on its RCP resistance and corroborate if the toughening effect that has been extensively reported with the addition of nanoclays under quasi-static and impact tests, remains under RCP conditions.

**Figure 1:**
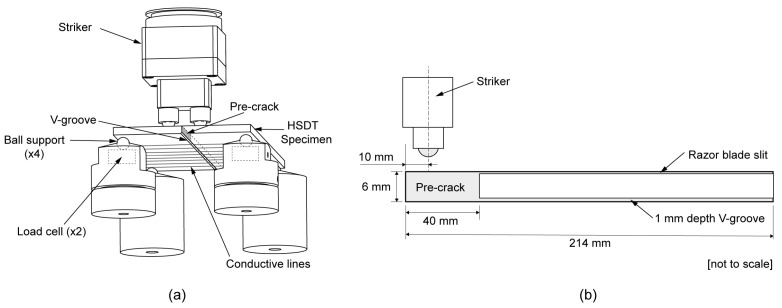
(**a**) The high-speed double torsion (HSDT) test configuration and (**b**) the specimen.

Different techniques have been developed to determine the dynamic fracture toughness of polymers, some of them based on the split Hopkinson pressure bar [14] or in dynamically loaded three-point bending specimens [15,16], both in combination with high-speed cinematography for capturing real-time crack initiation and propagation. Fond & Schirrer [17] and Kopp et al. [18] carried out dynamic fracture tests on strip band specimens to explore the brittle behavior of rubber toughened PMMA during rapid crack propagation. In this study, however, the high-speed double torsion (HSDT) test developed by Leevers & Williams [19] and Wheel & Leevers [20,21] will be used. This test is used to evaluate the dynamic fracture resistance under plane strain conditions ($G_{d1}$) in tough polymers by inducing rapid crack propagation at speeds of up to 350 m/s. The main advantages of this test are the long period of recordable crack growth, the curved crack front that promotes plane strain behavior in thin specimens, and the analytical simplicity of a geometry having only one degree of freedom: torsion [20]. Figure 1a describes the HSDT test configuration, in which a two-points striker impacts a polymer plaque resting horizontally on four support points, sending equal and opposite torsional waves along the two opposing halves of a V-grooved specimen, and promoting the rapid propagation of a crack along it.

In this study, the RCP resistance of different nanocomposites will be investigated through HSDT tests by recording crack speed, impact force and estimating $G_{d1}$, followed by a fracture surface analysis to identify the fracture macro- and micro-mechanisms that occurred during crack initiation and propagation. These results will be useful for understanding the effect of nanoclay addition on the dynamic fracture properties of HDPE nanocomposites and envisage potential applications in which nanoclay addition could prevent RCP.

## 2. Materials and Methods

### 2.1. Materials and HDPE–Nanoclay Composites Preparation

Polymer nanocomposites were produced using a commercial HDPE, Alathon 5618 from LyondellBasell, and a surface modified montmorillonite (MMT), Nanomer I.31PS nanoclay, which contains 15–35 wt% octadecylamine and 0.5–5 wt% aminopropyltriethoxysilane, supplied by Nanocor. A maleic anhydride grafted linear low-density polyethylene (MA-g-PE), OREVAC 18341 from Arkema, was used as a coupling agent. 

Initially, a matrix blend consisting of HDPE/MA-g-PE at a 10:1 weight ratio was prepared in a Beutelspacher SB-19 single screw extruder at 140 °C. The extruded material strand was cooled in a water bath and pelletized. HDPE–nanoclay composites were then prepared by incorporating 1, 3, and 5 wt% of I.31PS nanoclay into the HDPE/MA-g-PE matrix blend, designated as HDPE-1, HDPE-3, and HDPE-5, respectively. Prior to mixing, the nanoclays were sieved using a 20 μm mesh to remove large agglomerates and dried in a fan oven at 60 °C for 24 h. Nanocomposites were prepared in the same extruder at 140 °C, water cooled, and pelletized. The obtained HDPE–nanoclay composite pellets were extruded two more times to promote the shear-induced exfoliation of the nanoclays and a good dispersion in the HDPE/MA-g-PE matrix. To keep an equal thermal history, the same was done with the remaining HDPE/MA-g-PE (HDPE-0) pellets used as a control. 

For the HSDT tests, rectangular plates of 108 × 214 × 6 mm^3^ with a 1 mm depth 90 °V axial groove for guiding the crack path (Figure 1b) were molded in a Battenfeld HM 100/525 injection molding machine at an injection temperature of 190–220 °C, an injection pressure of 60 MPa, and a packing pressure of 50 MPa. The mold was kept at room temperature. A 40 mm pre-crack was introduced at one end of the plate (at the end opposite to the injection gate), while an axial razor blade slit was scored on the opposite surface of the V groove to inhibit ligament tearing [20].

### 2.2. Material Characterization Techniques

#### 2.2.1. Wide Angle X-ray Diffraction (WAXD)

Wide angle X-ray diffraction (WAXD) was performed on an A Xpert Pro diffractometer equipped with CuKα radiation (wavelength 0.1541 nm), operating at 40 kV, and 40 mA was used. Diffraction patterns were collected between 2.5° and 28° with a step size of 0.03° and a rate of 0.02°/min. A suitable sample of the I.31PS nanoclay was prepared by mounting and pressing the clay into an aluminum holder with a glass back support. Nanocomposite samples were injection-molded into 53 × 45 × 2.5 mm^3^ plates.

#### 2.2.2. Melt Flow Index (MFI)

A Dynisco LMI-5000 melt flow indexer was used to obtain the MFI of the different nanocomposites according to ASTM D1238. A mass of 0.68 kg was used at 190 °C. Three replicates for each nanocomposite were used.

#### 2.2.3. Dynamic Mechanical Analysis (DMA)

Complex modulus and damping properties were measured using a Q800 Dynamic Mechanical Analyzer. Injection-molded prismatic samples of 40 × 12 × 3 mm^3^ were loaded under the single cantilever bending mode. Scans were performed over a temperature range from −20 to 20 °C under a controlled sinusoidal strain (0.1–0.3 mm), at a frequency of 1 Hz and a heating rate of 5 °C/min.

#### 2.2.4. High-Speed Double Torsion (HSDT)

An aluminum striker with two steel ball bearings of a 10 mm diameter separated 25 mm from each other, was released and its speed monitored by a system of transmitter-receptor infrared sensors. The impact force was registered by a pair of piezoelectric load cells (PCB Model 208C) allocated under the two frontal ball bearings where the specimen rests (Figure 1). To monitor crack speed along the specimen, eight conductive lines perpendicular to the crack propagation direction (Figure 1a) consisting of silver ink traces and copper tape were delineated along the plate at 20 mm intervals, with the first line at 50 mm from the load plane. These lines worked as on/off switch circuits. As the crack propagated, the conductive lines were broken one by one, and the on/off transition at each line was detected and registered by digital counters configured at 100 MHz internal base clock, providing a 10 ns resolution. By knowing the time elapsed between the on/off switch of each pair of adjacent conductive lines, seven crack speed values were obtained, and the mean value for each HSDT test was reported. A NI-USB 6341 multifunction I/O device was used for data acquisition.

HSDT tests were performed at −5 °C and at a striker speed of 2.6 m/s. Once the specimen had been fractured, the raw data collected from the force and striker speed sensors, as well as the data corresponding to the crack propagation speed, were processed in LabVIEW and Matlab to estimate $G_{d1}$. Three replicates for each nanocomposite were used. 

#### 2.2.5. Stereo Microscopy and Scanning Electron Microscopy

The fracture surface characterization of the nanocomposites was performed using an LGA-MDG17 stereoscope and a JEOL−6360LV scanning electron microscope (SEM). All SEM specimens were sputter-coated with a thin layer of gold before observation to eliminate charging. 

## 3. Results and Discussion

### 3.1. Wide Angle X-ray Diffraction

**Figure 2:**
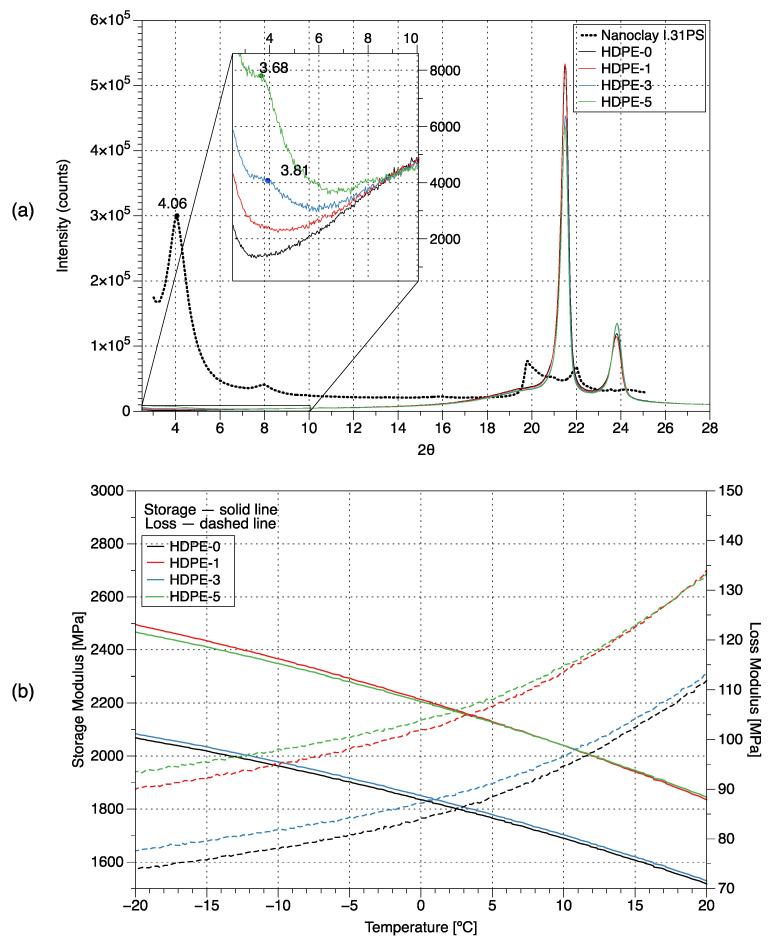
(**a**) The WAXD pattern and (**b**) storage and loss moduli for different HDPE–clay nanocomposites (containing 0, 1, 3, and 5 wt% of nanoclay, respectively).

In Figure 2a the wide angle X-ray diffraction (WAXD) patterns for the nanoclay and nanocomposites are shown. The (001) basal reflections were used to obtain the interlayer spacing of the nanoclay, whereas the (002) basal reflections of the nanocomposites were used to obtain the interlayer spacing of the clays in the HDPE-g-MA matrix. The results are summarized in Table 1. A d-spacing of 2.17 nm was found for the nanoclay, which agrees with reported values [22] for this nanoclay between 1.8 and 2.2 nm. The pattern for the HDPE-1 nanocomposite (containing a 1 wt% of nanoclay) was almost featureless, similar to the one of HDPE-0, suggesting exfoliation of the nanoclay, as has been previously reported [23], or at least intercalation to give an interlayer spacing greater than the measurement limit of the WAXD. 

The diffraction patterns for the HDPE-3 and HDPE-5 nanocomposites each exhibited a distinct peak corresponding to the (002) reflection. This appears as a shoulder on the main small angle peak in Figure 2a, as shown in the enlargement, due to the relatively small nanoclay content added to the polymer. The presence of this peak indicates an intercalated microstructure, where polymer chains are present between the clay layers, increasing the interlayer spacing compared to the pristine nanoclay (Table 1). Note that HDPE-3 shows a smaller interlayer spacing than HDPE-5, indicating a lower degree of intercalation. Although a specific type of surface-treated silicate may form an exfoliated structure at a low percentage inclusion in a polymer matrix, when larger concentrations are used the silicate may have insufficient room to exfoliate fully, and hence a more intercalated structure may be formed.

As described in [24,25], the relative crystallinity for each of the nanocomposites was obtained from the WAXD patterns by calculating the area under the curve of the (110) and (200) peaks corresponding to the crystalline structure and dividing it by the total area under the curve. Results are also included in Table 1. As observed, the relative crystallinity decreased with the increasing nanoclay content as was previously reported by [23], who explained that the nanoclays act as nucleating agents that increase the crystallization rate, promoting the formation of more spherulites but with a smaller size, resulting in a lower crystallinity. In addition, it is well known that the thermal conductivity of nanocomposites increases with the nanoclay content [26], resulting in higher cooling or solidification rates and thus lower crystallinity.

### 3.2. Melt Flow Index

Melt flow index (MFI) results are summarized in Table 1. The MFI decreases with the nanoclay content, indicating a higher viscosity when nanoclays are added. This is as expected because the nanoclays impede the movement of the polymer chains in the melt, e.g., [27], and increasing the volume fraction of the nanoclay will provide more impediment. The MFI decreases approximately linearly with the wt% of the nanoclay. 

### 3.3. Dynamic Mechanical Analysis

In Figure 2b the storage (E’) and loss (E’’) moduli of the nanocomposites measured using dynamic mechanical analysis (DMA) are shown, and results at −5 °C are summarized in Table 1. As observed, the addition of nanoclays increases both moduli, however, for HDPE-3 the increase is not as significant as for the other nanocomposites. The HDPE-1 nanocomposite has the highest storage modulus which can be related to its exfoliated/intercalated structure. It has been previously reported that when there is an effective nanoclay–polymer matrix interaction, the storage modulus increases with the clay content [28]. However, it has also been reported that at high amounts of clay content E’ starts to decrease, which has been related to a decrease in the d-spacing and to the inevitable presence of inhomogeneities or clay agglomerations [29]. This suggests that the HDPE-1 and HDPE-5 nanocomposites have a better interfacial interaction between phases than the HDPE-3 nanocomposite, which will be further corroborated through SEM analysis, as the presence of agglomerates may explain the behavior of the HDPE-3 nanocomposite.

A slight increase in the tan δ (E’’/E’ ratio) is observed for the HDPE-1 (0.7%), HDPE-3 (4.8%) and HDPE-5 (5.0%) nanocomposites. This ratio describes the damping of the material, which could be associated with the restraint or attenuation of the torsional stress waves during the HSDT tests.

### 3.4. High-Speed Double Torsion Tests

For the HSDT tests, the measured striker speed, mean striker force, and mean crack propagation speed, $\dot{a}$, are summarized in Table 2. Equation (1) was used to estimate the dynamic fracture resistance in plane strain condition, $G_{d1}$.

(1) $$G_{d1}=\frac{M^{2}}{\mu KB_{c}}\left(1-{\left(\frac{\dot{a}}{C_{T}}\right)}^{2}\right)$$

Where M is the moment generated by the striker force, μ is the shear modulus, K is a tabulated function of the beam cross section defined as $K=ZHB^{3}$, where H = 108 mm is the width of the specimen, B = 6 mm is the thickness of the specimen, and Z is a geometric factor defined as $Z=\frac{1}{3}-0.24\left(\frac{B}{H}\right)+0.13{\left(\frac{B}{H}\right)}^{2}$, $B_{c}$ = 5 mm is the fracture surface thickness calculated as B minus the groove depth of 1 mm, and $C_{T}$ is the torsional wave speed equal to 240 m/s [20]. Values of μ for each nanocomposite were estimated with the relation E=2μ(1+ν), where ν is the Poisson’s ratio. It was assumed that E’ = E, and that ν decreases with the nanoclay content. The HDPE matrix has a Poisson’s ratio $\nu_{0}\;$ = 0.43 [30], whereas a decrease in ν of 0.52% ($\nu_{1}\;$ = 0.42), 8.91% ($\nu_{3}\;$ = 0.39), and 24.03% ($\nu_{5}\;$ = 0.32) was estimated through numerical homogenization [31] for the HDPE-1, HDPE-3, and HDPE-5 nanocomposites, respectively. Estimated μ values were: 665 MPa, 808 MPa, 686 MPa, and 860 MPa for HDPE-0, HDPE-1, HDPE-3, and HDPE-5, respectively. The $G_{d1}$ results are also summarized in Table 2 and their mean values are included in Table 1 for comparison with other data. 

As observed, $\dot{a}$ decreases and $G_{d1}$ increases with the addition of nanoclays, however, as for DMA results, for HDPE-3 the effect is not as significant as for the other nanocomposites. At this point it is assumed that nanoclays are acting as crack deflection sites [7] which lower $\dot{a}$ and thus increase $G_{d1}$, but this will be further analyzed in Section 3.5 through the fracture surface analysis. Note also that the force measured by the piezoelectric sensors at the support decreases in the nanocomposites (−16%, −27%, and −20% for HDPE-1, HDPE-3, and HDPE-5, respectively). Although the external work in all the specimens was the same (equal striker mass and speed), the force registered by the sensors was different as each nanocomposite dissipated energy in different forms. The external energy input is distributed in three components during an increase in the fracture area during the RCP: the change in the energy required for crack propagation, change in the stored energy, and change in the kinetic energy due to inertial effects (important when the load changes abruptly or the crack grows rapidly). At this point it seems as if HDPE-3 dissipated the highest amount of energy, but not necessarily in crack propagation. Stored energy is affected by E’, for which HDPE-1 and HDPE-5 showed a significant increase. However, for HDPE-3 (containing 3 wt% of nanoclay) the E’ value remained almost unchanged, indicating a higher dissipation through elastic strain energy for the HDPE-3 nanocomposite. Because tan δ (damping) is similar for the three nanocomposites, a similar amount of energy dissipation through kinetic energy is expected, and thus the surplus energy that HDPE-3 dissipated through elastic strain energy must have been dissipated by the HDPE-1 and HDPE-5 nanocomposites during crack propagation, explaining their higher $G_{d1}$ values.

**Table 2:** HSDT test results for the different HDPE–clay nanocomposites (containing 0, 1, 3, and 5 wt% of nanoclay, respectively).

Specimen	Test Number	Mean Striker Force [N]	Striker Speed [m/s]	Crack Propagation Speed [m/s]	$G_{d1}$ [kJ/m^2^]
HDPE-0	1	131	2.6	207	0.14
	2	145	2.7	211	0.15
	3	150	2.7	201	0.21
	Mean	142 ± 10	2.7 ± 0.1	206 ± 5	0.17 ± 0.04
HDPE-1	1	136	2.7	172	0.24
	2	113	2.6	150	0.21
	3	109	2.6	145	0.26
	Mean	119 ± 15	2.6 ± 0.1	156 ± 14	0.23 ± 0.03
HDPE-3	1	101	2.6	144	0.20
	2	107	2.7	181	0.15
	3	104	2.7	162	0.19
	Mean	104 ± 3	2.7 ± 0.1	162 ± 19	0.18 ± 0.03
HDPE-5	1	103	2.7	86	0.26
	2	127	2.7	124	0.29
	3	111	2.6	79	0.27
	Mean	114 ± 12	2.7 ± 0.1	96 ± 24	0.28 ± 0.02

### 3.5. Fracture Surface Analysis

**Figure 3:**
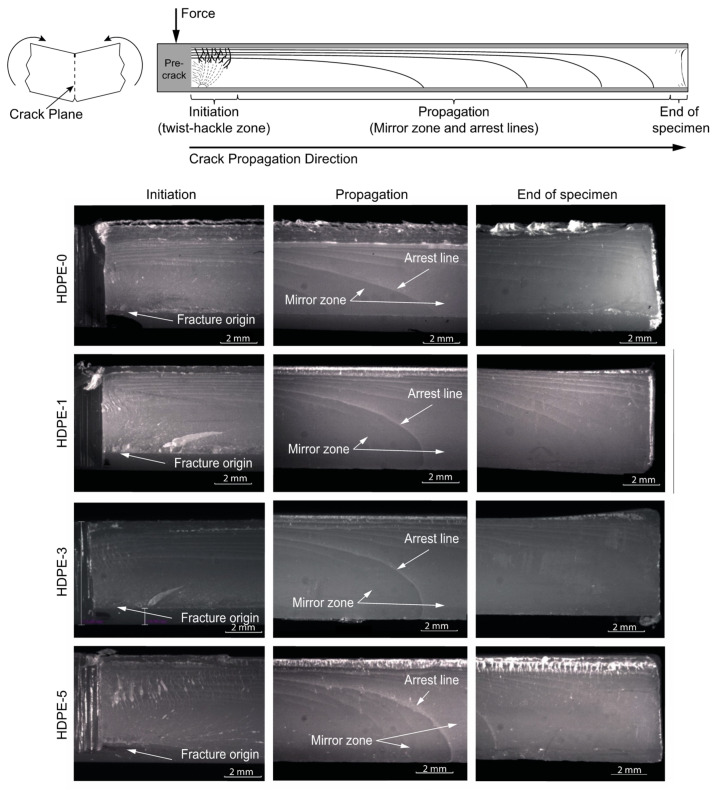
The initiation, propagation, and end-of-specimen fracture zones during RCP in HSDT test specimens for different HDPE–clay nanocomposites (containing 0, 1, 3, and 5 wt% of nanoclay, respectively).

Fracture is a multi-scale phenomenon as it depends on different energy dissipation mechanisms, manifested at different dimension levels, in the material structure during crack evolution [32]. The following analysis focuses on the fracture macro- and micro-mechanisms of the HDPE nanocomposites. Figure 3 shows the fracture surfaces of the different nanocomposites at a low magnification. As shown in the illustration, the RCP fracture surface produced by HSDT testing can be divided in three zones: initiation, propagation, and end-of-specimen.

Initiation and propagation zones are characterized by different plastic deformation mechanisms that play an important role in the evolution of the crack, absorbing different amounts of energy, and thus promoting acceleration or deceleration of the propagating crack [33]. These deformation mechanisms will also be affected by the nanoclay content. At this scale some basic features are observed. For the initiation zone, a river pattern growing from the fracture origin is observed, whereas for the propagation zone, white arrest lines are evident (Figure 3). According to [33] an arrest line does not represent an instantaneous position, but the locus of arrest points along a propagating front. When a crack propagates fast, stress waves propagate through the material and reflect off free surfaces (e.g., specimen boundaries), thus the conjunction of loading and unloading stress waves travelling in opposite directions can produce localized deceleration of the crack front, which, as it propagates, leaves an arrest line with a leading and trailing edge. Note that these arrest lines change from a sharper, more acute front for the HDPE-0 specimen to a blunter nose-shaped one for the nanocomposites (HDPE-1, HDPE-3, and HDPE-5), which agrees with their slightly higher damping (tan δ from DMA) and attenuation of stress waves. At the end-of-specimen, partial arrest lines that approach, but do not reach, the lower surface boundary are observed. In addition, for the HDPE-0 and HDPE-5 specimens, rougher surfaces at the upper boundary are observed when compared with the HDPE-1 and HDPE-3 specimens in which much flatter surfaces at the upper boundaries are visible. According to [33], this roughening at the upper boundaries corresponds to either very low or very high oscillatory crack speeds along the trailing edge of the crack front, which agrees with the measured crack speeds for these specimens: the highest for HDPE-0 and the lowest for HDPE-5. 

**Figure 4:**
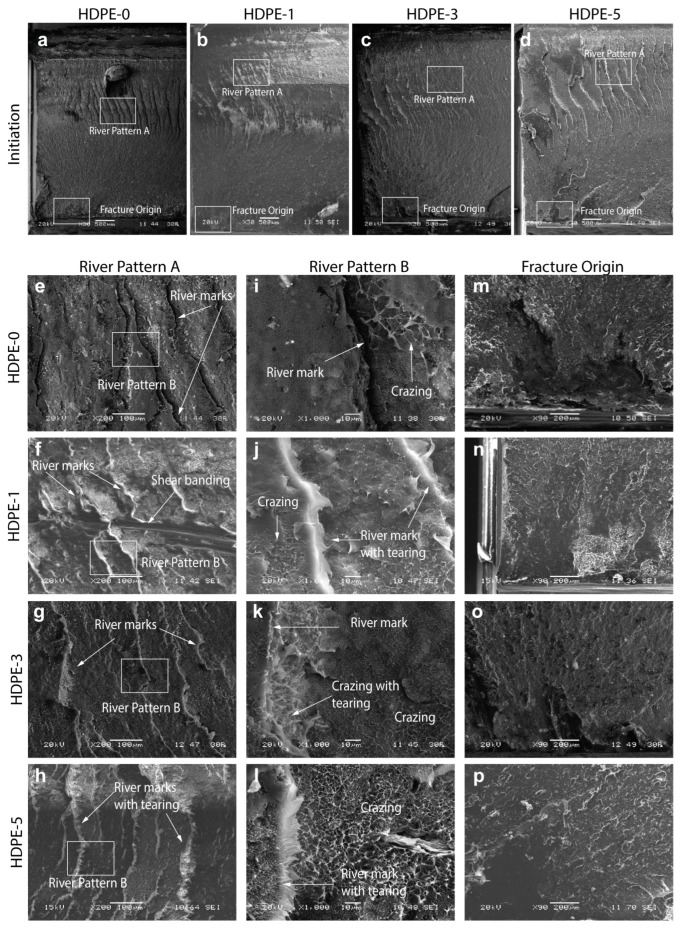
SEM images of the (**a**–**d**) initiation zone during RCP in the HSDT test specimens for different HDPE–clay nanocomposites (containing 0, 1, 3, and 5 wt% of nanoclay, respectively), and magnifications at (**e**–**h**) river pattern A, (**i**–**l**) river pattern B, and (**m**–**p**) the fracture origin.

Figure 4 shows the initiation zone for each nanocomposite observed using SEM. The fracture surface at the initiation zone (Figure 4a–d) is characterized by a well-reported river pattern [34,35,36] in which crack growth is stable, as the energy release rate is lower than the crack resistance. This pattern has also been referred to as hackle for samples fractured by bending and torsion [37] and described as a feathery or a leaf-like structure [37,38]. This zone is formed by the interaction of secondary crack fronts (which are either pre-existing or nucleated flaws growing stably in different directions and planes [39]) with the main crack front. The river lines grow from the fracture origin (bottom) towards the opposite (top) side, being rougher at the top side. Because of the torsional nature of the HSDT tests, the upper side is in compression (closing the crack rather than driving it through the material) while the bottom is in tension. However, as the crack propagates from the lower to the upper edge it produces an upward shift of the neutral axis, generating a sufficient tensile stress for crack growth [40]. Because the upper side presented more resistance to crack propagation it resulted in a rougher appearance. As observed, the rougher area increases with nanoclay content, suggesting that at the initiation zone, there is a greater significant energy absorption in the nanocomposites than in the pure HDPE.

A closer look at the river patterns (Figure 4e–h) shows how these river lines are out of the plane due to the torsional nature of the test, which makes the secondary cracks and the main crack interact in different angle planes. Moreover, shear banding is revealed for the HDPE-1 nanocomposite. These shear bands have been reported to be a plastic deformation mechanism which involves large amount of energy absorption in polymers when subjected to compressive stresses [41]. Crazing is observed in all specimens (Figure 4i–l), and this increases in density with the nanoclay content. For the nanocomposites tearing is also evident, which also increases with nanoclay content. Figure 4m–p shows in detail the fracture origin region from which the river marks grow. Interestingly, this region seems more brittle for the HDPE-1 and HDPE-5 nanocomposites, which might be explained by the fact that at the onset of fracture, the higher modulus of these nanocomposites promoted a more brittle behavior at the highly tensile region.

**Figure 5:**
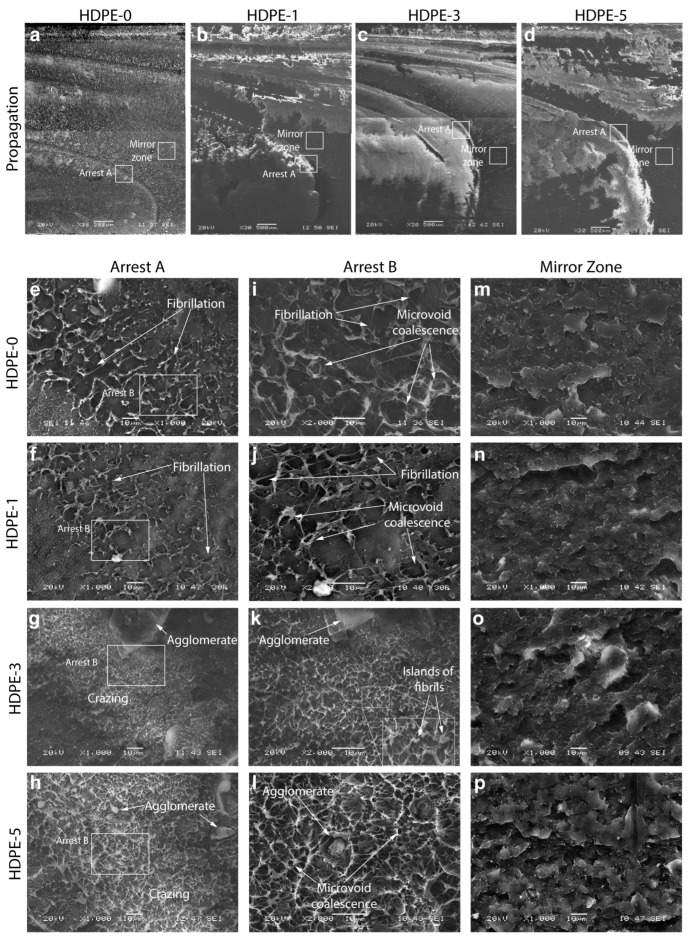
SEM images of the (**a**–**d**) propagation zone during RCP in the HSDT test specimens for different HDPE–clay nanocomposites (containing 0, 1, 3, and 5 wt% of nanoclay, respectively), and magnifications at (**e**–**h**) arrest zone A, (**i**–**l**) arrest zone B, and (**m**–**p**) the mirror zone.

Figure 5a–d shows the propagation zone for each nanocomposite observed by SEM. The propagation zone consists mainly of two distinct subzones: mirror zones and arrest lines. Arrest lines are well-defined whitening lines where crazing dominates, while the mirror zone is the brittle surface between arrest lines. The mirror zone has been defined as a brittle surface with the appearance of mosaic flakes which tends to be flat, meaning that the crack propagation velocity in this zone is higher than in any other zone [42].

Crazing is observed in the arrest zones of all specimens (Figure 5e–l), with significant fibrillation (extensively deformed fibrils) and microvoid coalescence dominating in the HDPE-0 and HDPE-1 specimens. Microvoid coalescence is a consequence of the extensive stretching of the polymer chains that are pulled into the fibrils of the craze, allowing neighboring microvoids to be coalesced [34]. Again, it is observed that crazing increases with the nanoclay content. This was expected as crazing occurs in the amorphous phase of the polymer [43], which increases with the nanoclay content (crystallinity decreases with the nanoclay content as seen in Table 1). Agglomerates of the nanoclays can be observed, especially for HDPE-3 and HDPE-5 specimens. Note that although nanoclays were sieved through a 20 μm mesh before adding them to the polymer, agglomerates larger than 20 μm in size are observed (Figure 5g). It has been widely reported that nanoparticles can be easily agglomerated when added or incorporated into the polymer matrix [44]. The formation of agglomerates could have either beneficial or detrimental effects. On the one hand, agglomerates facilitate crazing initiation in the sense that they provide sites for void nucleation, thereby lowering the stress required for crazing formation and thus inducing it at a larger extent [34]. However, if such agglomerates are found in the crystalline phase, they act as stress concentrator sites promoting premature failure. Note that the agglomerates in HDPE-3 are larger than those in HDPE-5, which agrees with studies that reported that an increase in volume fraction typically leads to smaller agglomerate sizes [44]. Because of the tendency of nanoparticles to agglomerate has been attributed to the direct mutual attraction between nanoparticles via van der Waals forces or chemical bonds, the difference in agglomerate size between the HDPE-3 and HDPE-5 nanocomposites can be explained as follows: it is assumed that for the HDPE-5 nanocomposite, in which the distance between the nanoclays is reduced, the agglomerates are denser (more compacted) and stronger; whereas in the HDPE-3 nanocomposite the nanoclays are loosely combined (less compacted) in the agglomerate [44], which might also explain the not-so-significant increase in mechanical properties for the HDPE-3 nanocomposite. In addition, in the HDPE-3 arrest zones, islands of fibrils are observed (Figure 5k), denoting a low fibril deformation and thus a lower energy consumption through plastic deformation. 

Finally, the characteristic flake appearance for the mirror zone is also observed in Figure 5. The toughening effect due to the addition of the nanoclays in a polymer matrix has been attributed to crack deflection and plastic deformation around the nanoparticles [7]. The crack deflection model proposed by Faber and Evans [45] assumes that as the crack approaches a particle in a composite material, it is deflected by the particle and follows another path direction. This effect is referred to as a shielding effect [46], with the new path direction being controlled by secondary cracks (growing some distance apart from the matrix–particle interface) which work as an attractor for the main crack tip, resulting in coalescence after the occurrence of a complex interaction between the corresponding stress fields [46]. Crack deflection will increase the roughness of the surfaces through tilting and twisting of the crack front [7], increasing the fracture surface area and thus increasing the fracture energy of the material. Figure 5m–p qualitatively suggest that the roughness of the surface increases with the clay content, which agrees with the $G_{d1}$ values reported for the HDPE-1 and HDPE-5 nanocomposites. This could be further corroborated through AFM roughness measurements. The increase in the fracture energy is due to the mixed mode of fracture that occurs during crack deflection due to a local modification of the propagation mode, from pure mode I (opening) to mixed mode I/II (opening/sliding) [47].

**Figure 6:**
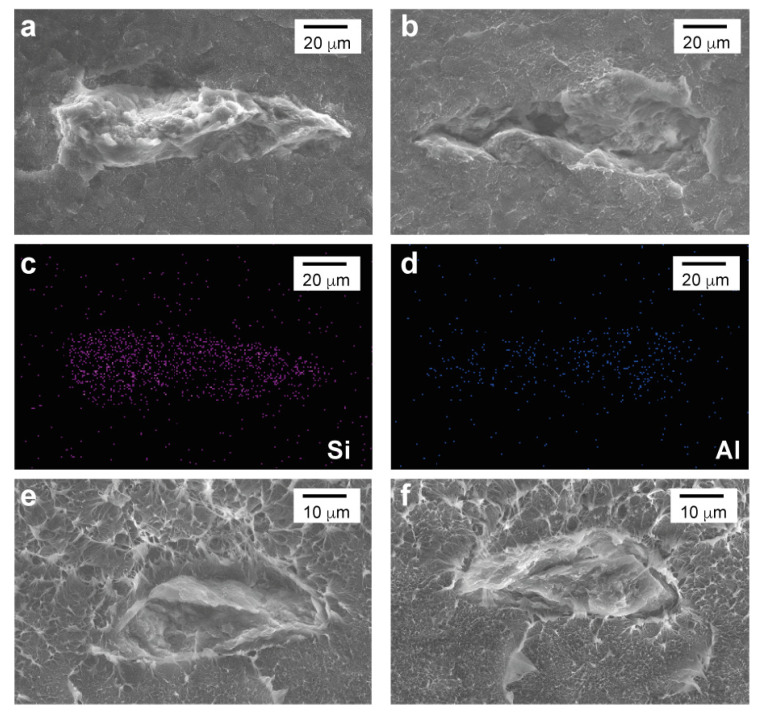
Agglomerate debonding and fracture on the HDPE-3 nanocomposite (containing a 3 wt% of nanoclay).

Faber and Evans also concluded that the aspect ratio of the particles has a significant effect on the toughening mechanism of particulate composites [45], which might explain the performance of the HDPE-3 nanocomposite in which large agglomerates were observed. In addition, the shielding effect mentioned above is dependent on the bonding strength between the particle (or agglomerate) and the matrix through its interface [46]. If the bonding is poor, debonding might occur. In Figure 6, debonding of an agglomerate in HDPE-3 is observed. On one side the agglomerate is shown (Figure 6a), and in the opposite side (Figure 6b) the cavity left by the agglomerate is evident. Energy-dispersive X-ray spectroscopy (EDS) confirmed that it was a nanoclay agglomerate as Si and Al were detected (Figure 6c,d). Debonding occurs due to a mismatch in the elastic and fracture properties between the HDPE chains and the nanoclay agglomerate [46], breaking the bonds that keep them temporally together (denoting a weak interfacial strength between them). 

On the other hand, when there is a high interfacial adhesion between the matrix and the particle (or agglomerate), but poor molecular interaction forces between the nanoclays, agglomerate fracture can occur. In Figure 6e,f, an agglomerate fracture is observed. The fractured agglomerate is observed on both sides of the fracture surface. This fracture is consistent with HDPE-3 having the lowest d-spacing, meaning that the coupling effect of the maleic anhydride was not achieved, resulting in a poor molecular interaction between the nanoclays. The fact that both phenomena (debonding and fracture) appeared in the HDPE-3 nanocomposite also contributed to the fact that $G_{d1}$ in this nanocomposite does not increase as much as HDPE-1 and HDPE-5.

## 4. Conclusions

The dynamic fracture resistance under plane strain conditions, $G_{d1}$, of HDPE-nanoclay composites was investigated through the high-speed double torsion test. The nanocomposites, with 1, 3, and 5 wt% of organo-silicate clays (HDPE-1, HDPE-3, and HDPE-5), showed an increase in $G_{d1}$ of 40%, 8%, and 65%, respectively. A similar enhancement trend was found for the viscoelastic properties (storage and loss moduli). 

Relative crystallinity estimated through the XRD patterns showed a decrease in crystallinity with clay content, indicating that the clays acted as nucleating agents that increased the crystallization rate, promoting smaller spherulites that resulted in lower crystallinity. This increase in the amorphous phase with the nanoclay content was consistent with the increase in crazing (and thus energy absorption) with the nanoclay content which was observed by SEM. However, the presence of large agglomerates in the HDPE-3 nanocomposite affected its performance, as agglomerate fracture and debonding was found on the microscopic examination of its fracture surface. For HDPE-1 large fibrillation and microvoid coalescence predominated as energy absorption mechanisms. XRD analysis suggested an exfoliated, or at least intercalated, structure for this nanocomposite. The HPDE-5 nanocomposite presented the highest improvement in mechanical properties, with crazing as the main deformation mechanism during fracture. These results show that exfoliated or intercalated nanoclays improve the RCP resistance of HDPE and could be considered in applications in which the rapid propagation of a crack must be prevented.

## Figures and Tables

**Table 1:** Nanocomposite d-spacing and relative crystallinity obtained from WAXD analysis, viscoelastic properties obtained using DMA, and $G_{d1}$ obtained using the HSDT test.

Sample	d-Spacing [nm]	Crystallinity [%]	MFI [g/10 min]	E’ @−5°C [MPa]	E’’ @−5°C [MPa]	tan δ @−5°C (×10^−2^)	Gd1 [kJ/m^2^]
HDPE-0	N/A	59.1	3.5 ± 0.1	1903	80	4.20	0.17 ± 0.04
HDPE-1	Exfoliated/Intercalated	58.9	3.3 ± 0.1	2294	97	4.23	0.23 ± 0.03
HDPE-3	2.32	57.2	2.6 ± 0.2	1908	84	4.40	0.18 ± 0.03
HDPE-5	2.40	55.9	2.1 ± 0.1	2270	100	4.41	0.28 ± 0.02

Note: d-spacing of pristine nanoclay is 2.17 nm.

## Data Availability

The data presented in this study are available on request from the corresponding author.
